# Divergent clinical outcomes of alpha-glucosidase enzyme replacement therapy in two siblings with infantile-onset Pompe disease treated in the symptomatic or pre-symptomatic state

**DOI:** 10.1016/j.ymgmr.2016.11.001

**Published:** 2016-11-18

**Authors:** Takashi Matsuoka, Yoshiyuki Miwa, Makiko Tajika, Madoka Sawada, Koichiro Fujimaki, Takashi Soga, Hideshi Tomita, Shigeru Uemura, Ichizo Nishino, Tokiko Fukuda, Hideo Sugie, Motomichi Kosuga, Torayuki Okuyama, Yoh Umeda

**Affiliations:** a Showa University Northern Yokohama Hospital, Children Medical Center, Yokohama, Kanagawa, Japan; b Showa University Northern Yokohama Hospital, Cardiovascular Center, Yokohama, Kanagawa, Japan; c Department of Neuromuscular Research, National Institute of Neuroscience, National Center of Neurology and Psychiatry, Kodaira, Tokyo, Japan; d Department of Pediatrics, Hamamatsu University School of Medicine, Hamamatsu, Shizuoka, Japan; e Faculty of Health and Medical Sciences, Tokoha University, Hamamatsu, Shizuoka, Japan; f Division of Medical Genetics, National Center for Child Health and Development, Setagaya-ku, Tokyo, Japan; g Department of Clinical Laboratory Medicine, National Center for Child Health and Development, Setagaya-ku, Tokyo, Japan

## Abstract

Pompe disease is an autosomal recessive, lysosomal glycogen storage disease caused by acid α-glucosidase deficiency. Infantile-onset Pompe disease (IOPD) is the most severe form and is characterized by cardiomyopathy, respiratory distress, hepatomegaly, and skeletal muscle weakness. Untreated, IOPD generally results in death within the first year of life. Enzyme replacement therapy (ERT) with recombinant human acid alpha glucosidase (rhGAA) has been shown to markedly improve the life expectancy of patients with IOPD. However, the efficacy of ERT in patients with IOPD is affected by the presence of symptoms and cross-reactive immunologic material (CRIM) status.

We have treated two siblings with IOPD with ERT at different ages: the first was symptomatic and the second was asymptomatic. The female proband (Patient 1) was diagnosed with IOPD and initiated ERT at 4 months of age. Her younger sister (Patient 2) was diagnosed with IOPD at 10 days of age and initiated ERT at Day 12. Patient 1, now 6 years old, is alive but bedridden, and requires 24-hour invasive ventilation due to gradually progressive muscle weakness. In Patient 2, typical symptoms of IOPD, including cardiac failure, respiratory distress, progressive muscle weakness, hepatomegaly and myopathic facial features were largely absent during the first 12 months of ERT. Her cardiac function and mobility were well-maintained for the first 3 years, and she had normal motor development. However, she developed progressive hearing impairment and muscle weakness after 3 years of ERT. Both siblings have had low anti-rhGAA immunoglobulin G (IgG) antibody titers during ERT and have tolerated the treatment well.

These results suggest that initiation of ERT during the pre-symptomatic period can prevent and/or attenuate the progression of IOPD, including cardiomyopathy, respiratory distress, and muscle weakness for first several years of ERT. However, to improve the long-term efficacy of ERT for IOPD, new strategies for ERT for IOPD, e.g. modifying the enzyme to enhance uptake into skeletal muscle and/or to cross the blood brain barrier (BBB), will be required.

## 1 Introduction

Pompe disease, also known as glycogen storage disease type II (OMIM 232300), is an autosomal recessive, lysosomal storage disorder caused by acid α-glucosidase (GAA; OMIM 606800) deficiency, resulting in the rapid accumulation of lysosomal glycogen in skeletal, cardiac, bulbar, and smooth muscles [1], [2]. Pompe disease is classified into infantile-onset form and late-onset forms based on organ involvement, age of onset, and rate of disease progression. Infantile-onset Pompe disease (IOPD) is characterized by massive deposition of glycogen in multiple organs, including heart, liver and skeletal muscle, resulting in rapidly progressive cardiomyopathy, hepatomegaly, generalized muscle weakness, respiratory insufficiency, hypotonia, and motor delay. Cardiorespiratory involvement subsequently leads to significant functional impairment and a very limited lifespan. Death from cardiac and/or respiratory failure generally occurs by age 2 [3], [4].

Enzyme replacement therapy (ERT) with recombinant human acid alpha-glucosidase (rhGAA, alglucosidase alfa, Myozyme®, Sanofi Genzyme, Cambridge, MA) is available for the treatment of Pompe disease [5], [6]. ERT has been shown to dramatically improve the prognosis and quality of life of patients with IOPD. Early initiation of ERT in IOPD reduces the risk of death and invasive ventilation in the first year of life. To achieve the optimal outcome, ERT should be started before symptoms are apparent and irreversible damage has occurred [7], [8]. Newborn screening (NBS) is the best way to diagnose patients and enable initiation of treatment in a timely manner in the pre-symptomatic period [9], [10]. NBS for Pompe disease has already started in Taiwan and in several US states, and it has been successful in improving the prognosis of patients with IOPD [11], [12].

Several cases of ERT-treated siblings with IOPD have been reported [13], [14]. Two cross-reactive immunologic material (CRIM)-negative siblings receiving ERT had good clinical outcomes in the setting of low anti-rhGAA immunoglobulin G (IgG) antibody titers [15], [16]. Here, we report our treatment experience in two Japanese sisters with IOPD. The older sister (Patient 1) was diagnosed symptomatically and started treatment at 4 months of age, whereas the younger sister (Patients 2) was diagnosed pre-symptomatically based on the positive family history and started treatment at 12 days of life. As expected, the treatment response and quality of life differed significantly between the sisters. Early diagnosis and initiation of ERT led to a better clinical outcome. However, long-term follow-up shows that current ERT has limitations with respect to efficacy in IOPD.

### 1.1 Case report

#### 1.1.1 Patient 1 (older sister)

Patient 1 was the second child born to unrelated Japanese parents whose previous daughter was healthy. Birth weight was 3534 g at 40 weeks gestation. One-minute Apgar score was 8. Patient 1 was admitted to the NICU for the first 5 days of life due to mild cyanosis and failure to thrive. Her symptoms improved, and she was discharged to home. Creatinine kinase (CK) at birth was 2121 IU/L. At 4 months of life, she developed a barking cough and stridor at rest and was hospitalized with a suspicion for croup. Body weight was 7.1 kg (+ 0.6 SD) and length was 67 cm (+ 1.5 SD). Heart rate was 101 beats per minute and had a regular rate and rhythm, and blood pressure was 81/34 mm Hg. Heart sounds were muffled without a murmur or third or fourth sounds. Respiratory rate was slightly increased to 34 breaths per minute, and stridor was audible over both lung fields. Oxygen saturation on room air was 95%. The liver was palpable to 6 cm below the right costal margin and 4 cm below the midline, whereas the spleen was not palpable. She had head lag, and her legs were in a frog-like position. She had generalized hypotonia and decreased active movements.

**Fig. 1:**
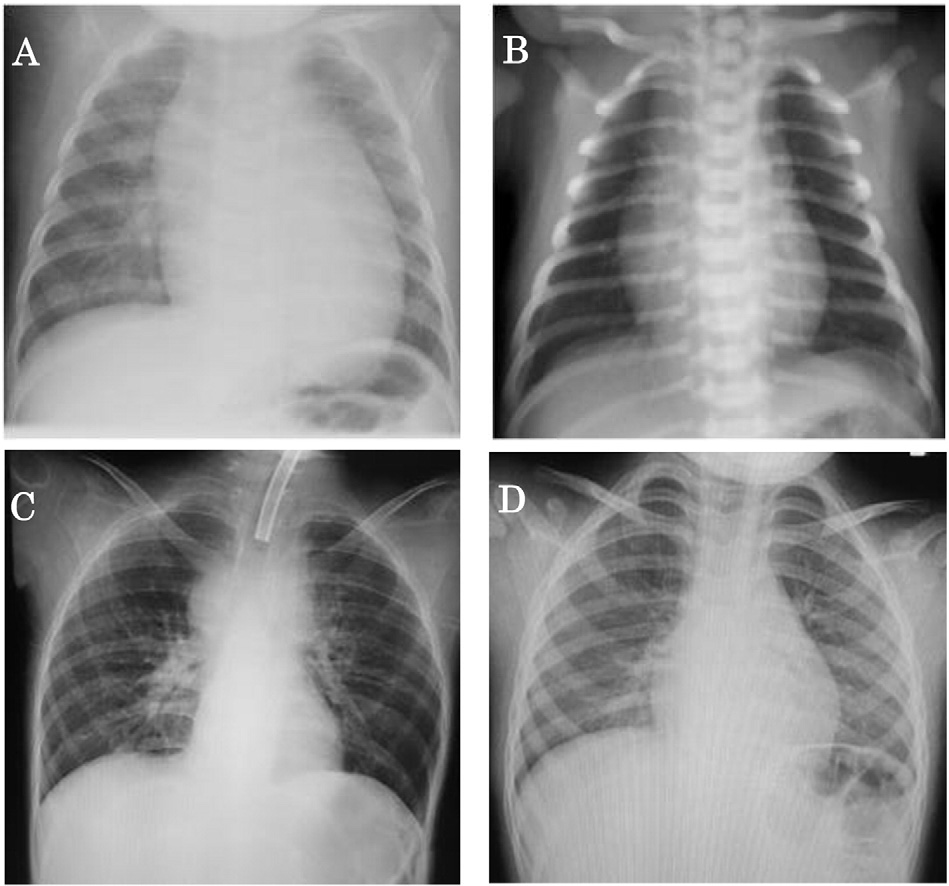
Chest X-ray before and after ERT in two siblings. A. Patient 1 (older sister) at 4 months of age before ERT. Cardiomegaly present (cardiothoracic ratio 65%). B. Patient 2 (younger sister) at birth before ERT. No cardiomegaly present. C. Patient 1 at 7 years of age and after 6 years and 8 months of ERT. No cardiomegaly present. D. Patient 2 at 3 years and 4 months of age and after 3 years and 4 months of ERT. No cardiomegaly present.

**Fig. 2:**
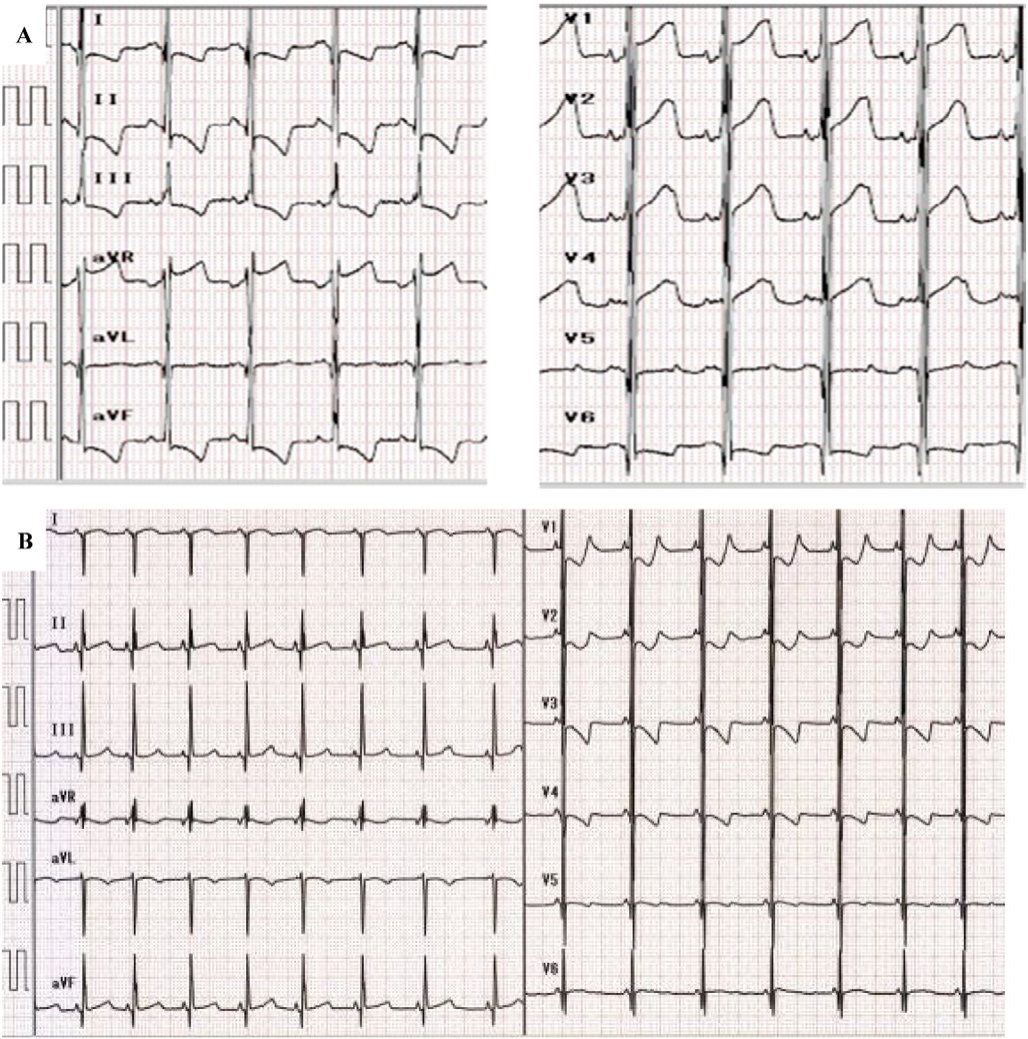
Electrocardiogram (ECG) at baseline in two siblings. A. Patient 1 at 4 months of age and before ERT. ECG shows short PR interval (0.10 s) and high voltage in all chest leads (maximum voltage of R + S was 16 mV in V2) with elevated ST changes in V1. B. Patient 2 at birth and before ERT. ECG shows short PR interval (0.08 s), high voltages (maximum voltage of R + S was 7 mV on V2), and inverted T waves from V1 to V4.

**Fig. 3:**
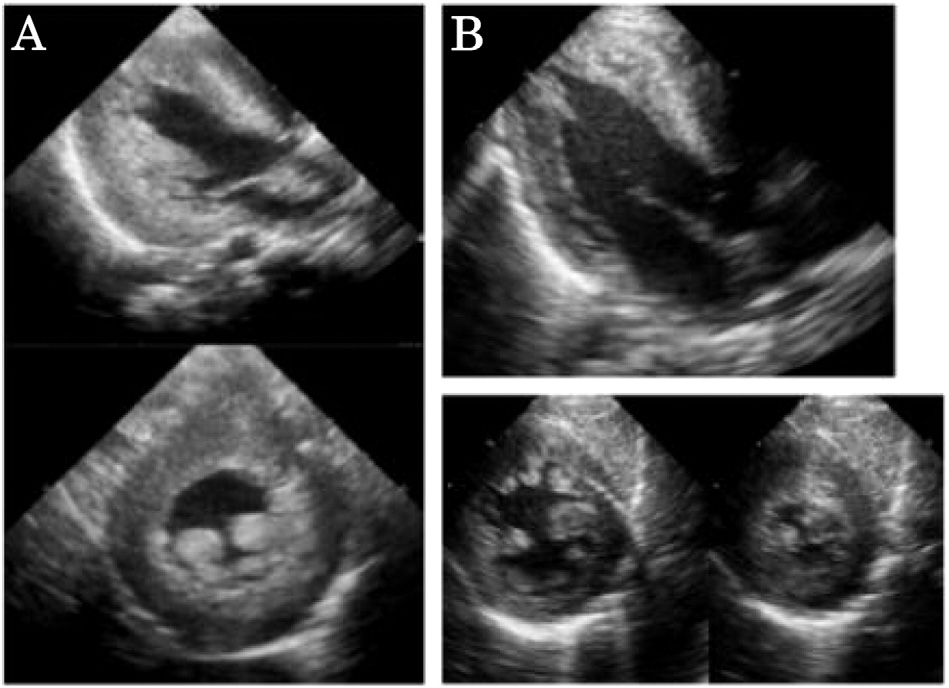
Echocardiography (ECHO) findings before ERT in two siblings. A. Patient 1 at 4 months of age and before ERT. ECHO shows remarkable left ventricular wall thickening with a left ventricular end-diastolic posterior wall thickness (PWd) of 14 mm (328% of normal) and an inter-ventricular septum end-diastolic thickness (IVSd) of 12 mm (307% of normal). The left ventricular end-diastolic dimension (LVIDd) was 25.8 mm (98% of normal), and there was no left ventricular outflow stenosis. B. Patient 2 at birth and before ERT. LVMI was 120 g/m^2^ (198% of normal), which was calculated by ECHO.

**Fig. 4:**
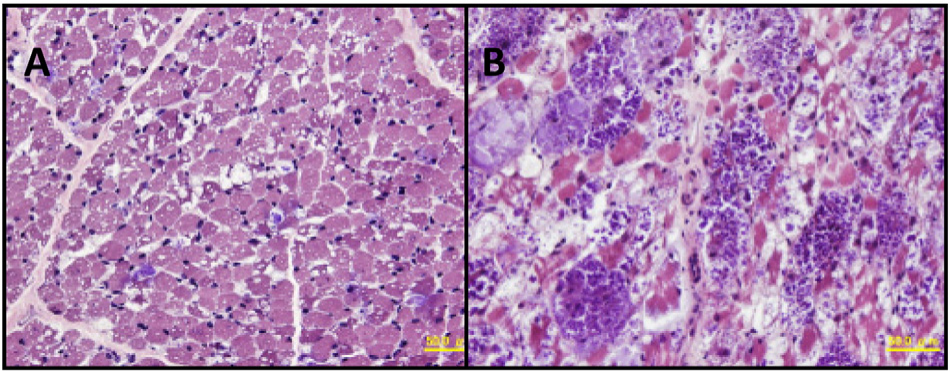
Muscle histopathology (H&E stain) in Patient 1 at the time of diagnosis (A) and 1 year after ERT (B). A: Scattered muscle fibers have the intracytoplasmic vacuoles of approximately 30 μm in diameter which are filled with basophilic amorphous materials. In addition, tiny intracytoplasmic vacuoles are seen in most fibers. B: Intracytoplasmic vacuoles with basophilic materials are much more prominent than A with the size of > 50 μm in diameter. Adipose tissue infiltration is also seen in part.

Chest X-ray showed cardiomegaly (cardiothoracic ratio of 65%) (Fig. 1A). Electrocardiogram (ECG) documented a short PR interval (0.10 s) and high voltages in all chest leads (maximum voltage of R + S was 16 mV in V2) with elevated ST changes in V1 (Fig. 2A). Echocardiography (ECHO) demonstrated remarkable left ventricular wall thickening: the inter-ventricular septum end-diastolic thickness (IVSd) was 12 mm and the left ventricular end-diastolic posterior wall thickness (PWd) was 14 mm. The left ventricular myocardium mass index (LVMI) was calculated to be 392 g/m^2^ by ECHO. On the other hand, the left ventricular end-diastolic dimension (LVIDd) was 25.8 mm (98% of normal), and there was no left ventricular outflow stenosis. The left ventricular ejection fraction (EF) was decreased to 47% (Fig. 3A). Skeletal muscle biopsy histopathology showed many enlarged lysosomes, approximately 30 μm in diameter, in muscle fibers also filled with storage material and vacuolated fibers (Fig. 4A, B). GAA activity was low at 0.15 nmol-4MU/min/mg in lymphocytes (Normal range: 5.6 ± 1.87 nmol-4MU/mg/min) and 0.1 nmol-4MU/mg/30 min (Normal range: 14.6 ± 4.8 nmol-4MU/mg/30 min) in a muscle biopsy specimen. Mutation analysis of the *GAA* gene revealed compound heterozygosity for a frameshift mutation, c.483dupC (p.K162QfsX15) (very severe), and a missense mutation, c.1696 T > C (p.S566P) (potentially less severe).

#### 1.1.2 Patient 2 (younger sister)

Patient 2, the third daughter of this couple, was born three years after Patient 1. The parents received genetic counseling during the pregnancy. She was born by vaginal delivery at 40 weeks gestation with a birth weight of 3442 g. There were no abnormal respiratory or motor findings at birth, and no cardiomegaly was present by chest X-ray (Fig. 1B). However, ECG demonstrated a short PR interval (0.08 s), high voltages (maximum voltage of R + S was 7 mV on V2), and inverted T waves from V1 to V4 (Fig. 2B). Cardiac hypertrophy was detected by MRI. LVMI was 130 g/m^2^ (198% of normal) by ECHO (Fig. 3B). CK was elevated to 2898 IU/L (Normal range: 40–310 IU/L) at birth. GAA activity in lymphocytes on Day 10 of life was low at 0.64 nmol/mg protein/h (Normal range: 30.7 ± 10.3 nmol/mg protein/h). Patient 2 had the same *GAA* genotype as her older affected sister.

#### 1.1.3 ERT treatment and clinical assessment

Enzyme replacement therapy (ERT) with recombinant human acid alpha- glucosidase (rhGAA, alglucosidase alfa, Myozyme®; Sanofi Genzyme, Cambridge, MA, USA) was initiated in Patient 1 at 4 months of age and in Patient 2 at 12 days of age at a dose of 20 mg/kg diluted in saline solution and administered intravenously every other week.

Laboratory and physical examinations were performed every 12 weeks. Standard 12-lead ECG and ECHO were performed at Baseline and every 12 weeks. Liver and spleen volumes were estimated by palpation. Serum was collected every 12 weeks and sent to a central laboratory (Sanofi Genzyme, Framingham, MA, USA) for evaluation of anti-rhGAA IgG antibodies. The two sisters have received ERT for 9 years (Patient 1) and 5 years (Patient 2). Both experienced only a single mild infusion-associated reaction (erythema) at their initial rhGAA infusions. Treatment was well-tolerated in both patients.

## 2 Results

### 2.1 Cardiac function

Although no ECG was performed at birth in Patient 1, similar ECG abnormalities were observed in both siblings before initiation of ERT and at all assessments thereafter, which consisted of a short PR interval, high voltages, and ST-wave abnormalities. Patient 1's ECHO revealed moderately increased LVPd and IVSd, papillary muscle and tendon thickening, normal LVIDd, and decreased EF. During the first 12 weeks of treatment, LVMI decreased significantly and there was recovery of the EF. Patient 2's LVMI was increased at birth and decreased following ERT.

### 2.2 Respiratory findings

Patient 1 had a barking cough, stridor, and slight tachypnea at baseline. Oxygen saturation on room air was 95%. Subsequently, she developed recurrent respiratory infections and ventilatory disturbance prompting the initiation of biphasic cuirass ventilation and supplemental oxygen at 1 year of age. Rapid progression of respiratory failure ensued, requiring invasive mechanical ventilation with tracheostomy 9 months later. Patient 2 has had no abnormal respiratory findings at 5 year of age.

### 2.3 Neurologic findings

**Fig. 5:**
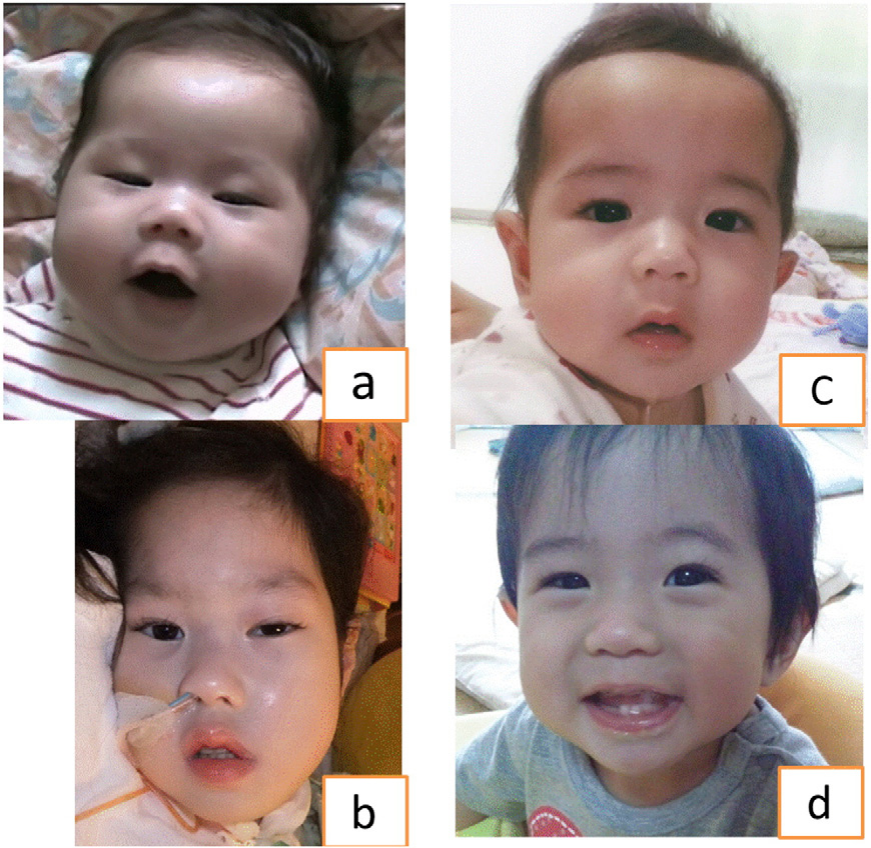
Development of facial muscle weakness over time in Patient 1 (a, b) and Patient 2 (c, d) with infantile-onset Pompe disease treated with ERT. Comparison at 4 months of age (a, c) with their most recent photographs (b, d). Facial muscle weakness, present in Patient 1 at both ages, is absent at both ages in Patient 2. Patient 1 and Patient 2 initiated ERT at 4 months and 12 days of age, respectively.

**Fig. 6:**
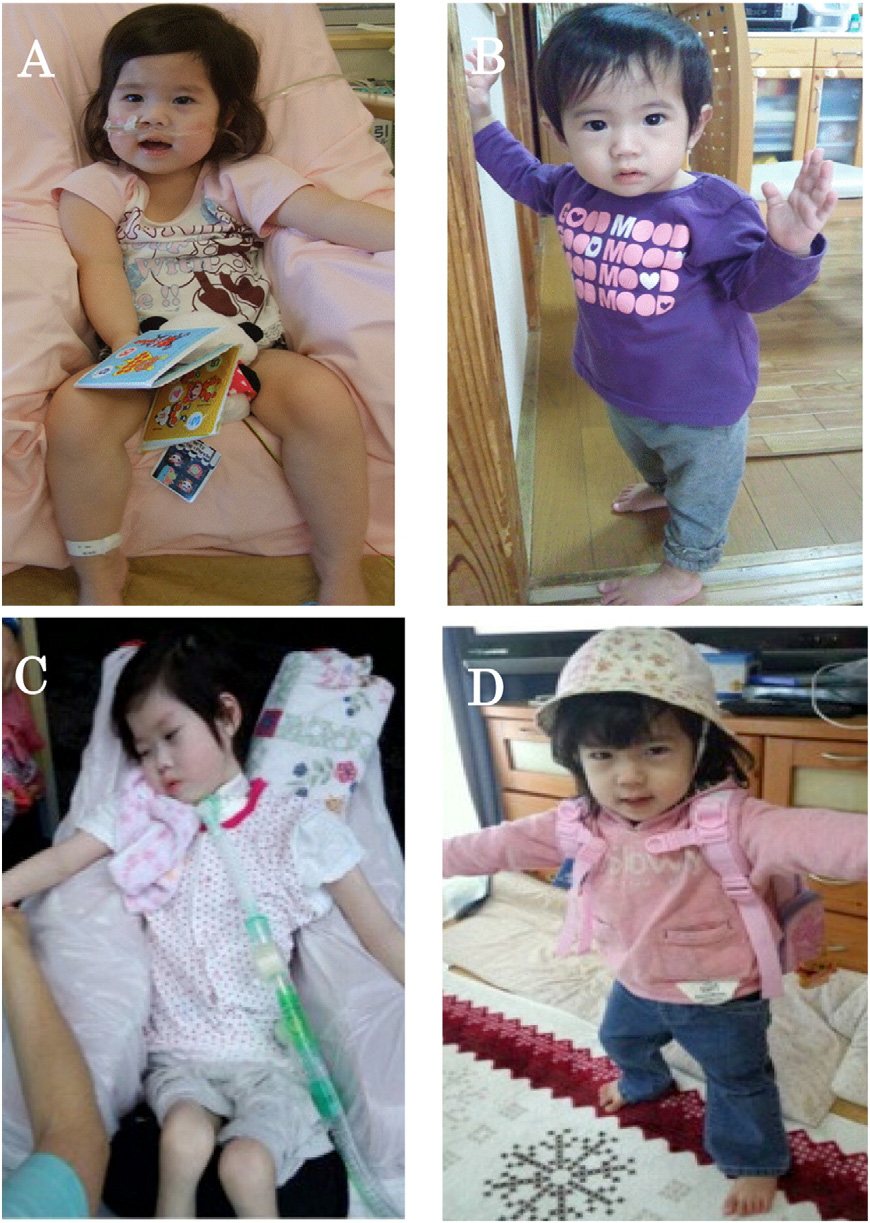
These figures show the motor development of the sisters. A. Patient 1 (older sister) required neck and back supports when sitting at 1 year old. B. Patient 2 (younger sister) had normal development and could stand alone at 1 year old. C. Patient 1 was bedridden and required invasive ventilation for 24 h due to the gradual progression of muscle weakness at 6 years old. D. Patient 2 had independent walking at 1 year of age and achieved normal developmental milestones without cardiorespiratory failure through 3 years of age.

Patient 1 had muscle weakness before initiation of ERT. She also had ptosis, a minor nasolabial fold, and sunken cheeks consistent with facial hypotonia (Fig. 5a). Motor development was severely delayed. As she was unable to hold her head up, she required head and back support while sitting at 1 year of age (Fig. 6A). Despite ERT, her muscle weakness progressed. She required tube feeding at 9 months of age and became bedridden at 2 years old (Figs. 5b, 6C). Patient 1 was only able to move her eyes, the corners of the mouth, and fingertips slightly. Brain MRI at 6 and 9 years old showed cerebral atrophy (data not shown).

In contrast to Patient 1, Patient 2 had a normal facial appearance at 4 months of age (Fig. 5c), was walking independently at 1 year of age (Fig. 6B), and had normal developmental milestones without cardiorespiratory failure through 3 years old (Figs. 5D, 6D). Developmental quotient (DQ) was assessed with the Tanaka-Binet Intelligence Scale. Her DQ was normal (DQ = 89) when assessed at 3 years and 1 month. However, after 3 years of ERT she developed a waddling gait, a positive Gower's sign, dysarthria and slight hearing impairment. Her DQ was getting worse (DQ = 72–73) when assessed at 5 years and 6 month. Table 1 compares the findings in Patient 1 at baseline and after 1,6 and 9 years of ERT, to Patient 2 at baseline and after 1,3 and 5 years of ERT.

### 2.4 Muscle histopathology

Skeletal muscle was biopsied for histopathological examination in Patient 1 at the time of diagnosis (A) and after 1 year of ERT (B). On hematoxylin and eosin (H&E) stain, the lysosomal vacuoles were much larger in size in B than A, suggesting increased storage. Adipose tissue infiltration was also seen in part in B.

### 2.5 Laboratory examinations

High serum CK levels were present at birth in both sisters. Over the course of the study, the CK levels decreased with treatment but remained elevated in both sisters. CK levels in Patient 1 at baseline and after 1,6 and 9 year of ERT were 1051, 1014, 275 and 56 IU/L, respectively. CK levels in Patient 2 at birth and after 1, 3 and 5 year of ERT were 2898, 377, 1406 and 1489 IU/L, respectively. l-aspartate aminotransferase (AST) and l-alanine aminotransferase (ALT) levels were also high at birth and elevated AST and ALT remained after ERT initiation in both sisters.

### 2.6 Serum anti-rhGAA IgG antibodies

At baseline, both sisters were seronegative for anti-rhGAA IgG antibodies. Both developed serum anti-rhGAA IgG antibodies by 3 months. The peak antibody titer in Patient 1 was 6400 after 3 months of ERT and in Patient 2 was 400 after 7 months of ERT. Subsequently, both patients showed declining anti-rhGAA IgG antibody titers that remained low during ERT.

## 3 Discussion

It is well-recognized that ERT with rhGAA dramatically improves the prognosis and quality of life of patients with IOPD. This positive clinical outcome suggested that rhGAA treatment effectively and rapidly reduces glycogen accumulation in the heart [5], [7], [8]. As cardiorespiratory failure is one of the primary causes of death in IOPD, the rapid reduction of myocardial hypertrophy in all patients after initiation of rhGAA treatment almost certainly contributes to their prolonged survival. Cardiac failure or EF calculated by ECHO was rapidly restored to normal function by ERT. Myocardial hypertrophy was observed in both sisters in this study; however, LVMI calculated by ECHO was not reduced to normal levels in the first year of ERT in Patient 1.

Compared to its effect on cardiac failure, ERT's effect on muscle weakness varies between individual patients, depending on when ERT is started after the emergence of symptoms. Muscle function may not recover completely, even if glycogen accumulation in skeletal muscle is reduced, because the amount of glycogen in skeletal muscle and motor function are not proportional [17]. In addition, CRIM-negative status and/or the presence of high and sustained neutralizing anti-rhGAA IgG antibodies may prevent improvement in muscle weakness.

A previous study evaluated muscle biopsies from 8 IOPD patients treated weekly with 10 mg/kg of rhGAA at baseline and at 12 and 52 weeks post-treatment as a pharmacodynamic indicator of efficacy. Glycogen clearance varied widely among these samples, which correlated well with clinical outcomes. Low glycogen levels, mild ultrastructural damage, a high proportion of type I fibers, and young age at baseline were associated with a good histologic response [18]. Untreated IOPD is characterized clinically by early death in infancy, whereas with ERT survival is prolonged. However, muscle weakness is improved only when ERT is initiated in the asymptomatic state or at an early age. Consequently, NBS offers the best opportunity to improve the prognosis of this otherwise fatal disease [7].

As for other inborn errors of metabolism, NBS for Pompe disease enables early diagnosis and initiation of treatment in the pre-symptomatic period. The NBS program for Pompe disease has already started in Taiwan and in several US states, and it has successfully improved the prognosis of patients with IOPD. In Japan, NBS for Pompe disease is not generally available except for in a few hospitals and local areas [19]. Patient 2 reported in this paper was evaluated for IOPD because of her affected older sister. She was diagnosed by the finding of low GAA activity by dried blood spot (DBS) analysis at 10 days of life, whereas the levels of GAA activity on days 1 and 5 were borderline. Accordingly, early treatment with ERT was able to start in the pre-symptomatic period at 12 days of age, although there were signs of cardiac hypertrophy. Considering the difference in the clinical outcomes between the two sisters, one who had started rhGAA ERT in the presence of symptoms (Patient 1) and the other in their absence (Patient 2), it seems warranted to initiate ERT, whenever possible, at early age before symptoms are apparent.

In Taiwan, a national NBS program for Pompe disease was started in 2005. By 2011, approximately 470,000 newborns had been screened, and 10 patients were diagnosed with IOPD. These 10 patients were started on ERT at a median age of 21.6 days (range 6 to 34 days) and were followed for a median duration of 63 months. At last follow-up, all patients were alive and free of mechanical ventilation. Left ventricular hypertrophy improved and was stable thereafter. However, many patients developed orofacial manifestations, including facial muscle weakness, hyper-nasal speech, ptosis, and hearing loss [20]. Their mean anti-rhGAA IgG antibody titer was 1230 (range 0–6400). Later, a second Taiwanese group conducted a NBS program for Pompe disease and found that even earlier administration of ERT in asymptomatic IOPD patients was associated with better outcomes [21]. In this study, 14 of 669,797 newborns screened were diagnosed with IOPD, including 13 who were diagnosed after 2010 when rapid screening became available. These 13 patients initiated ERT at a mean age of 11.9 days (range 6 to 23 days) and were treated for a mean duration of 2.7 years (range 1.1–5.3 years). All maintained normal cognitive function and motor function, and none had hearing loss or abnormal vision. The mean age for independent walking (11.9 months) was normal. Anti-rhGAA IgG antibody titers were very low in all patients (mean 584, range 0–3200), and they declined after year 1 in 9 of 10 patients and became seronegative in three patients. The results of these two Taiwanese NBS studies suggest that earlier initiation of ERT in the pre-symptomatic period can influence the immune response and the long term outcomes of patients with IOPD. In our study, only two patients with IOPD were reported. However the patients are sisters from the same family. They have same background factors including gene, environment, ethnic group and sex so on. This fact suggests that in our case the confounding factors are less than the other reports and the difference in the clinical outcomes between the two sisters depends simply on early initiation of ERT in the pre-symptomatic period.

Patient 2 was diagnosed with IOPD at 10 days of age and initiated ERT at 12 days of age. Although the CRIM status of this patient was unknown, the presence of a missense mutation might have allowed some GAA protein production, given the sisters' low anti-rhGAA IgG antibody titers over several years of ERT. Another possibility is that early initiation of treatment, before the immune system has matured, allowed for natural immune tolerance. Based on the early initiation of treatment and low antibody titers, Patient 2 was predicted to be a good responder. While the clinical outcome of Patient 2 was much better than that of Patient 1, Patient 2 eventually developed hearing loss and moderate hypotonia. Our case report suggests that early diagnosis and initiation of ERT during the pre-symptomatic period can prevent and attenuate the progression of IOPD, including cardiomyopathy, respiratory distress and muscular weakness for first several years of ERT. However, to improve the long-term efficacy of ERT for IOPD, new strategies for ERT for IOPD, e.g. modifying the enzyme for more efficient uptake into skeletal muscle or crossing the blood-brain barrier (BBB), will be required.

## Figures and Tables

**Table 1:** Comparison of clinical signs and symptoms between elder sister and younger sister.

	Patient 1 (elder sister)				Patient 2 (younger sister)			
	Before ERT	After ERT			Before ERT	After ERT		
Age	4 months	1 year	6 years	9 years	At birth	1 year	3 years	5 years
Cardiac condition	Failure	Fair	Good	Good	Normal	Normal	Normal	Normal
Hepatomegaly	6 cm on subcostal arch poor limb motion	Not palpable	Not palpable	Not palpable	1 cm on subcostal arch	Not palpable	Not palpable	Not palpable
Muscular weakness	Poor limb motion Head lag Frog leg position	Fair finger motion Lower limbs shake slowly	Loss of automatic limb movements	Loss of automatic limb movements	Normal	Normal	Normal	Gowers' sign(+) need wheel chair for long walking
Respiratory condition	Failure	Oxygen administration extrathoratic respirator	Invasive ventilation with tracheostomy	Invasive ventilation with tracheostomy	Normal	Normal	Normal	Normal
Facial muscular weakness	Slight smile responsively	Smile responsively	Slight smile responsively	Slight movement of the corners of the mouth	Crying naturally	Smile	Smile	Smile
Ptosis	Present	Not present	Not present	Not present	Not present	Not present	Not present	Not present
Nasolabial fold	Present slightly	Present slightly	Absence	Absence	Present	Present	Present	Present
Sunken cheeks	Present	Present	Present	Present	Present	Present	Present	Present
Motor development	Poor head control	Sitting with neck and back support	Bedridden	Bedridden	Normal	Stand alone	Running and jumping	Wadding gait
Speaking	Crying for a short time	Single syllables for a short time	Unable to speak	Unable to speak	Crying naturally	Babbling	Normal	slurred speech
Hearing	Moderate deaf (60 dB–70 dB +)	Moderate deaf (50 dB–60 dB +)	Severe deaf (80 dB–90 dB +)	Unevaluated	Normal	Normal	Moderate deaf (50 dB–60 dB +)	Mild deaf (20 dB–45 dB +)
Developmental quotient (DQ)	–	Unevaluated	Unevaluated	Unevaluated	–	–	89 (At the age of 4 years)	72–73
Brain MRI finding	Normal	NA	Slight bilateral frontal cerebral atrophy, chronic subdural hematoma	Slight bilateral frontal cerebral atrophy	Normal	NA	Normal	NA
Electrocardiogram (maximum R + S voltage)	16 mV on V2	7.5 mV on V3	11.5 mV on V3	9.8 mV on V3	8.4 mV on V1	6.3 mV on V2	8.1 mV on V3	6.7 mV on V3
LVMI (g/m^2^)	392	95	106	59	130	78	68	102
EF (%)	47	73	90	84	81.7	74.5	76.6	78.6
IVSd (mm)	12.2	4.5	8.5	6.8	5.9	5.4	5.6	6.7
LVPWd (mm)	14.5	6.9	9.6	6.5	4.3	4.7	3.4	5.0
LVIDd (mm)	25.8	30.4	24.1	36.5	24.5	32.2 (At the age of 2 years)	34.1	39.0
CK	1051	1014	275	56	2898	377	1406	1489
AST/ALT	229/116	192/149	51/58	32/24	132/28	91/65	195/121	250/129
Anti-rhGAA IgG antibody titer	Negative	800	N/A	100	Negative	100	200	Negative

CK: Serum creatine kinase, AST: l-aspartate aminotransferase, ALT: l-alanine aminotransferase.
